# The Otago Exercise Program: Innovative Delivery Models to Maximize Sustained Outcomes for High Risk, Homebound Older Adults

**DOI:** 10.3389/fpubh.2017.00054

**Published:** 2017-03-23

**Authors:** Tiffany E. Shubert, Lavinia Spring Goto, Matthew Lee Smith, Luohua Jiang, Holly Rudman, Marcia G. Ory

**Affiliations:** ^1^Center for Health Promotion and Disease Prevention, University of North Carolina, Chapel Hill, NC, USA; ^2^School of Physical Therapy, South College, Knoxville, TN, USA; ^3^Health Services Administration Program, University of Phoenix-Oregon Campus, Salem, OR, USA; ^4^Department of Health Promotion and Behavior, Institute of Gerontology, The University of Georgia College of Public Health, Athens, GA, USA; ^5^Department of Health Promotion and Community Health Sciences, Texas A&M School of Public Health, College Station, TX, USA; ^6^Department of Epidemiology, School of Medicine, University of California Irvine, Irvine, CA, USA; ^7^Northwest Senior and Disability Services, Salem, OR, USA; ^8^Department of Health Promotion and Community Health Sciences, Texas A&M School of Public Health, College Station, TX, USA

**Keywords:** Otago Exercise Program, fall prevention, frail, innovation, aging, health promotion, evidence-based

## Abstract

**Background:**

It is estimated one in two adults age 80 and over fall each year, resulting in substantial morbidity and mortality rates among this oldest-old population. The Otago Exercise program (OEP) is an evidence-based fall prevention program shown to reduce falls by 35% among high-risk older adults. The OEP was designed to be delivered in the home by physical therapists. This model has encountered multiple implementation challenges in the United States health-care system, which has resulted in the development and testing of innovative models to support a broader reach and dissemination of this program.

**Methods:**

The Northwest Senior and Disability Services is an Area Agency on Aging (AAA) serving a five-county region in Oregon. This AAA developed a model where a Certified Occupational Therapy Assistant (COTA) and exercise physiologist delivered the OEP with a physical therapist available to consult on all cases. Physical function assessments and self-reported perceptions about physical function were collected at baseline and 6 months.

**Results:**

Baseline measures were collected on 239 participants enrolled in the OEP, and 62 participants at 6 months. Those who completed 6 months of the OEP demonstrated significant improvements in all physical function assessments and self-perceived functional improvements. A subset of this group that demonstrated improvements in the ability to rise from a chair also reported significantly fewer falls during the 6-month intervention.

**Conclusion:**

Innovative models in which the OEP exercise sessions are delivered by non-physical therapists appear to be effective in improving physical performance measures and decreasing fall risk over a 6-month period. Because these models do not require a physical therapist, they may require fewer resources to implement. These findings have implications to inform implementation and dissemination strategies to bring the OEP to scale.

## Introduction

One out of three adults over age 65 fall each year (1), posing a significant impact on quality of life and a significant burden on the health-care system (2). Older adult falls are typically attributed to multiple risk factors such as leg muscle weakness, chronic diseases, and polypharmacy (too many or the wrong type of medications) (3). Older adults who have a greater number of risk factors are at a much higher likelihood of experiencing a fall and a fall-related injury (3).

NorthWest Senior and Disability Services (NWSDS) is an Area Agency on Aging (AAA) that serves a five-county region spanning over 4,500 mi^2^ in the Northwestern part of Oregon. This service area has the potential to reach a population of over 100,000 seniors and people with disabilities. Approximately, 30% of clients served are considered “dual-eligibles,” meaning they qualify for both Medicare and Medicaid services. The dual-eligible population has high rates of multiple chronic conditions and disability, which are indicators of increased fall risk (4, 5). This population also accounts for a disproportionate amount of health-care spending—20% of the Medicare population is composed of dual-eligibles, yet they account for 35% of all Medicare expenditures (6).

NorthWest Senior and Disability Services had identified that a large number of clients had multiple risk factors for falls. Several clients were experiencing multiple falls, fall-related injuries, and hospitalizations. Many seniors reported falls during health assessments conducted by NWSDS. Even though there were evidence-based fall prevention programs available in the community in Oregon (7), the most frail older adults were physically unable to take advantage of these classes, even when transportation was made available. This raised concern in the community, especially because frail older adults are most likely to experience multiple falls and fall-related injuries (8).

The high rate of falls and fall-related injuries was resulting in a significant impact on quality of life and financial burden to the state. To address this issue, NWSDS wanted to leverage the public health fall prevention initiatives in Oregon (9) with state health promotion dollars made available to AAA to deliver evidence-based programs directed at the high-risk clients (10). NWSDS determined the most effective programs were those delivered in the home. As such, the Otago Exercise Program (OEP) met these criteria and was selected as a potential solution. The OEP was developed and evaluated in New Zealand in the late 1990s. The original randomized controlled trials reported improvements in functional outcomes and a 35% reduction in falls for frail, high-risk older adults (11, 12). These results have been replicated in multiple studies in different settings (13–16). The OEP is recognized by the Centers for Disease Control and Prevention as an evidence-based fall prevention program (17), and the National Council on Aging has categorized OEP as meeting the highest level criteria for evidence-based programs (18).

The OEP consists of 5 warm up and 17 strength and balance exercises, which are progressed over the course of the plan of care. Examples of exercises include (with weights on the ankles): bending and straightening the knee from a sitting position, standing on one leg for 30 s, walking in a heel-toe pattern, and standing up and sitting down from a chair (19). The original program was designed for a physical therapy (PT) to work with an older adult client in their home for six visits over a 1-year period. The first four visits were in the first 2 months of the program (i.e., initial visit, a visit a week later, then 2 weeks, then 4 weeks), then follow-up visits were conducted at 6 and 12 months with monthly “check-in” phone calls during the course of the program (12). The PT selected appropriate exercises from the 17 and progressed the exercises for the participant over the course of the program. This model sets the stage for client engagement and ownership of their exercise program (the program only works if the client does the exercises). The OEP has achieved high levels of adherence with over 35% of participants stating they perform the exercises three times a week, 1 year after the start of the program (11, 12).

Although this model is highly effective, dissemination in the United States (US) has been limited (20). Challenges arise because the OEP is delivered at a much lower frequency of visits over a much longer duration than a typical PT episode of care. As such, documentation and billing practices have posed substantial barriers to the implementation of the OEP by PTs. As a result, a typical duration of the OEP in the US is 8 weeks as opposed to 6 months (19). This model is referred to as the US OEP model. In addition, PTs do not typically partner with AAA to implement programs (20).

NorthWest Senior and Disability Services was aware of the effectiveness of the OEP and interested in offering it to their clients. However, the limited availability of PTs and PTAs, and reimbursement challenges, made it difficult to implement the US OEP. NWSDS proposed to implement an innovative dissemination model, the Community OEP, which leveraged the resources available to serve as many clients as possible. The Community OEP used an experienced Certified Occupational Therapy Assistant (COTA) to screen and select appropriate OEP exercises, certified personal trainers to deliver the program, and a PT consultant to provide program oversight. The program was designed to be delivered in a slightly different frequency to the US OEP: three visits in the first 3 weeks of the program, then visits once a month for the next 5 months, for a total of eight visits in the first 6 months as opposed to five. The COTA or the personal trainer also called participants weekly for the first 6 months as opposed to monthly calls.

In this model, the PT can intervene with high-risk clients as appropriate, but the PT does not conduct one-on-one sessions or the phone calls. The PT is not constrained by billing and documentation practices because all providers—PT, COTA, and personal trainer, were hired by the AAA. This model allowed participants to complete a 6-month intervention.

The Community OEP can be offered as a long-term intervention to a frail population who could receive great benefit from the program. However, when introduced to this service area, it was unknown who would participate, if participants would continue for 6 months and if participants would achieve improvements in outcome measures associated with fall risk. Therefore, the purposes of this study were to analyze the data collected from this intervention to (1) describe the participants in the Community OEP program; (2) compare characteristics between Community OEP completers (i.e., with baseline and 6-month data) and non-completers (i.e., those with only baseline data); (3) describe outcomes of participants after 6 months; and (4) identify trends in falls and fall-related injuries based on functional performance during the 6-month intervention.

## Materials and Methods

### Community OEP Model

Participants were referred to the program by the NWSDS. Referrals were made by case workers, drivers of the Meals on Wheels Program, local Coordinated Care Organizations, community members, and family members. Referral criteria included being aged 65 years or older and having concerns related to the participant’s fall risk or mobility level.

### Procedures

Each participant referred received an evaluation by the COTA to determine appropriateness for the Community OEP. Participation criteria included able to walk safely with or without a device in the home, and able to perform the exercises on their own or with the help of a caregiver. If deemed appropriate, the participant signed all necessary paperwork to participate in this intervention offered by NWSDS, including a waiver of liability. During the initial evaluation, the COTA completed all baseline data collection, a home safety check, and a medication review.

After the evaluation, the COTA developed the exercise plan that was reviewed by the PT consultant. Recommendations from the consultant were incorporated into the plan, and the client was scheduled for the Community OEP visit #1. At this visit, the client was taught their OEP. Subsequent visits were performed by the personal trainer. All new and current cases were reviewed weekly with the PT consultant. In addition, the COTA and personal trainer had access to a nurse and a dietician on an as needed basis.

### Data Collection

This was a translational study of implementation; therefore, there were no specific inclusion or exclusion criteria for participants entered into the database. The only inclusion criterion was that participants needed to be prescribed the OEP and measures taken at baseline and 24 weeks.

All baseline and post-assessment data were collected by the COTA who administered questionnaires and functional tests. Questionnaire data included socio-demographic characteristics (e.g., age, sex, race, ethnicity), fear of falling (no, yes), and falls history (i.e., the number of falls they experienced in the past year, number of injuries, number of emergency room visits, and number of hospitalizations). Additional questions included self-reported health status (excellent, very good, good, fair, and poor), satisfaction with current activity levels (very, mostly, somewhat, or not at all), and confidence about their ability to keep themselves from falling (4-point scale ranging from strongly agree to strongly disagree) (21). Self-reported perceptions about functional ability were assessed by the reported level of difficulty in performing various activities (e.g., climbing one flight of stairs) on a four-point scale ranging from “no difficulty” (scored 1) to “unable to do” (scored 4) (22). Participants were also asked how often they restrict their activities because of difficulties in walking (always, sometimes, seldom, never).

Functional tests included the Timed Up and Go (TUG) test (23, 24), the 30-Second Chair Rise test (Chair Rise) (25, 26), and the Four-Stage Balance test (Four Stage) (27). Each of these tests has been validated to screen for increased risk of falls and functional decline and is part of a standard assessment for fall risk (28). The TUG measures the time needed to stand up from a standard arm chair, walk 3 m, turn around, return to the chair, and sit down again (29). Times greater than 12 s are indicative of increased risk of falling (28). The 30-Second Chair Rise requires the older adult to demonstrate the ability to stand from a standard height chair one time without using their arms. If successful, they are asked to stand up and sit down as many times as possible in 30 s without using their arms. Their score is compared to age- and gender-based normative values, with scores lower than average considered an increased risk for falling (28). The Four-Stage Balance Test requires the older adult to stand in progressively more challenging positions (Stage 1—feet side-by-side; Stage 2—one foot slightly in front of the other; Stage 3—heel-toe; and Stage 4—single leg stance) and hold each position for at least 10 s. Those that cannot hold either Stages 3 or 4 for at least 10 s are considered at increased risk of falling (28).

All measures were repeated at 6 months. Additional information collected included the number of PT visits (if any) and number of falls experienced during the program. The COTA recorded data on a paper copy and then entered the de-identified information into the database. The database was created and housed at UNC Chapel Hill. The COTA was responsible for entering in data at baseline, 8 weeks, 6 months, and discharge. The database automatically assigned an ID number. There were no unique identifiers or personal health information recorded in the database. This study was deemed exempt by the UNC Office of Human Research Ethics from Institutional Review Board.

### Statistical Analysis

Baseline characteristics were examined for all participants and compared to identify any significant differences between groups. Various analyses were performed to examine change from baseline to post-assessment for functional performance and perceived functional performance outcomes for each site. Linear mixed models (using SAS Proc Mixed procedure) were fitted for continuous outcome variables. Linear mixed effects models are likelihood-based approaches that use all available data in model estimation and provide unbiased estimates of the intervention effects under the assumption of missing at random. General Estimating Equation models with logit link function (using SAS Proc GENMOD procedure) were employed to examine changes from baseline to post-assessment for binary outcome variables. All the regression models included appropriate covariance structure to account for the correlation among repeated measures from the same participant. To eliminate any systematic bias and examine the direct effects of this intervention, regression analyses controlled for the participant’s age and sex as well as the number of falls they reported in the past 12 months, the number of weeks they received PT prior to beginning the OEP, and the delivery site where the client was reached. Falls data were collected *via* self-report at 8 weeks and at 6 months. The falls reported for these two time periods were combined to determine the number of falls, fall-related emergency room visits, and fall-related hospitalizations experienced by each participant during the 6-month intervention.

## Results

### Participant Characteristics

Table 1 provides baseline data characterizing the 239 participants engaged in the Community OEP, which as then compared by their completion status: non-completers (*n* = 177) and 6-month completers (*n* = 62). Overall, 70% of participants were female, and the majority was white. The majority (87%) reported a fear of falling, and 60% had experienced at least one fall in the past year. There were no significant differences in demographics, fear of falling, or falls history between completers and non-completers.

**Table 1:** **Baseline demographics and comparisons between non-completers and completers at 6 months**.

Baseline	Baseline (*n* = 239)			Baseline non-completers (*n* = 177, 74.1%)		Completers (*n* = 62, 25.9%)		Test statistic *X*^2^ or Fisher’s exact test or *t* test	*p* value for *X*^2^ or Fisher’s exact test or *t* test
		
	Total ***n***	*n* or mean	% or SD	*n* or mean	% or SD	*n* or mean	% or SD		
Age	239	79.82	11.8	80.53	11.59	77.79	12.13	1.58	0.12
Sex	239							0.96	0.33
Male		73	30.5	51	28.8	22	35.5		
Female		166	69.5	126	71.2	40	64.5		
Hispanic	239							N/A^a^	0.68
No		231	96.7	170	96.1	61	98.4		
Yes		8	3.4	7	4.0	1	1.6		
Race	239							N/A^a^	0.66
White		221	92.5	161	91.0	60	96.8		
Black or African-American		5	2.1	4	2.3	1	1.6		
Asian		2	0.8	2	1.1	0	0.0		
Others		11	4.6	10	5.7	1	1.6		
Fear of falling	203							0.10	0.75
No		26	12.8	19	13.3	7	11.7		
Yes		177	87.2	124	86.7	53	88.3		
Timed Up and Go (TUG)	215	26.2	22.7						
Low risk (enrollment TUG time <12 s)		29	13.5	22	75.9	7	24.1	0.18	0.67
High risk (enrollment TUG time ≥12 s)		186	86.5	134	72.0	52	28.0		
Chair stand	215	6.0	4.5						
Low risk (>average scores)		66	30.8	54	81.8	12	18.2	4.59	0.03
High risk (≤average scores)		148	69.2	100	67.6	48	32.4		
Fall in past year	208							0.0006	0.98
No		80	38.5	57	38.5	23	38.3		
Yes		128	61.5	91	61.5	37	61.7		
# of falls in past year	208	1.75	1.9	1.71	1.9	1.83	1.9	−0.43	0.67
# of falls resulting in injuries	205	0.59	1.0	0.58	1.0	0.62	1.1	−0.24	0.81
# of falls resulting in ED visits	204	0.26	0.6	0.23	0.6	0.33	0.6	−1.10	0.27
# of falls resulting in hospitalization	205	0.18	0.6	0.13	0.5	0.28	0.6	−1.79	0.08
# of weeks of physical therapy (PT) prior to Otago	197	1.43	3.0	1.75	3.2	0.66	2.3	2.37	0.02
# PT visits prior to Otago	197	2.72	6.0	3.42	6.4	1.07	4.4	2.57	0.01

*^a^Fisher’s exact test*.

Functional performance measures of the TUG and Chair Rise tests were obtained on 215 participants at baseline. At baseline, 86.5% of participants scored at risk category for the TUG test, and 69.2% scored at risk for the Chair Rise. When comparing completers versus non-completers, the groups were at similar risk for the TUG (*p* = 0.67). At baseline, a larger proportion non-completers were in the low-risk group for the Chair Rise (*p* = 0.03). On average, those in the completer group received significantly fewer weeks of PT prior to beginning the Community OEP (1.1 ± 4.4) compared to the non-completer group (3.4 ± 6.4). Similarly, those in the completer group received significantly fewer PT visits to beginning the Community OEP (0.66 ± 2.3) compared to the non-completer group (1.8 ± 3.2).

Of the 239 participants who started the program, pre-post functional performance and perceived functional performance data were collected on 57 participants (Table 2). Significant improvements were observed from baseline to the 6-month post-assessment the TUG test (*p* < 0.01) and the Chair Rise (*p* < 0.01). For the Four Stage, the proportion of participants who could achieve Stages 3 or 4 significantly increased (*p* < 0.01). For the perceived functional performance measures, the proportion of participants who reported excellent or very good health status, and felt confident they could keep themselves from falling significantly increased (*p* < 0.01). For the self-report functional ability measures, the proportion of participants that stated they had “no difficulty” performing all activities listed (*p* < 0.01), with exception of climbing one flight of stairs (*p* = 0.10).

**Table 2:** **Performance changes from baseline to 6-month post-intervention survey for program completers (*N* = 57)**.

	Baseline			Post-intervention (6 month)			Mean change from pre- to post-survey^a^	Odds ratio^b^	*p*^c^
					
	*N*	Mean	SD	*N*	Mean	SD			
**Functional performance**									
TUG times (seconds)	55	24.39	14.4	55	20.03	14.8	−4.36 (±8.39)^d^	–	<0.01
Chair stand	57	5.54	4.3	57	7.42	4.4	1.88 (±4.01)^d^	–	<0.01

		***n***	**%**		***n***	**%**			

Stages 3 or 4 in Four Stage	53	14	26.4	53	27	50.9	–	9.60	<0.01
**Perceived functional performance**									
Excellent or very good health status	57	11	19.3	57	20	35.1	–	14.32	<0.01
Very/mostly satisfied with physical activity levels	57	10	17.5	57	31	54.4	–	1.32	<0.01
Feel confident not falling (strongly agree or agree)	56	24	42.9	56	45	80.4	–	2.33	<0.01
No difficulty in walking across room	57	25	43.9	57	42	73.7	–	2.75	<0.01
No difficulty in walking one block	57	11	19.3	57	23	40.4	–	10.29	<0.01
No difficulty in stooping, crouching, kneeling	56	3	5.4	56	14	25.0	–	6.83	<0.01
No difficulty in getting out of a straight back chair	57	21	36.8	57	31	54.4	–	1.63	<0.05
No difficulty in climbing one flight of stairs	55	5	9.1	55	11	20.0	–	1.00	0.10
Never or seldom restrict activities because of difficulties in walking	57	14	24.6	57	24	42.1	–	5.17	0.02

*^a^Mean changes based on paired t-tests*.

*^b^Odds ratios from McNemar’s tests*.

*^c^p value from paired t-test for continuous variables and from McNemar’s test for binary variables*.

*^d^The Minimal Detectable Change (MDC) for the TUG (at 80% power and alpha = 0.05) is −3.0; the minimal detectable change for 30-Second Chair Rise (at 80% power and alpha = 0.05) is 0.9*.

Self-reported falls history, emergency room visits, and hospitalizations due to a fall were collected over the 6-month period. These data were verified by the COTA. Data were compared at 6 months between participants who scored at “high risk” and “low risk” for falls based on defined benchmarks for each functional assessment test: TUG, Chair Rise, and Four Stage. Significantly fewer falls were reported by participants who scored in the low-risk group for the Chair Rise test at 6 months (65 falls/participant versus 2.45, *p* < 0.01). No other significant differences were found in major events experienced by the 6-month completer group (Table 3).

**Table 3:** **Falls and fall-related injuries for completers based on functional performance at baseline**.

	*N*	falls		*p* value	#ED visits		*p* value	Hospitalization		*p* value
		Mean	SD		Mean	SD		Mean	SD	
Baseline functional performance										
**TUG times (s)**	**49**	**1.37**	**2.25**	**0.37**	**0.20**	**0.58**	**0.60**	**0.08**	**0.34**	**0.07**
Low risk (<12 s)	7	1.00	1.29		0.29	0.76		0.29	0.76	
High risk (≥12 s)	42	1.43	2.38		0.19	0.55		0.05	0.22	
**Chair stand**	**49**	**1.37**	**2.25**	**<0.01**	**0.20**	**0.58**	**0.85**	**0.08**	**0.34**	**0.22**
Low risk (>average)	11	0.27	0.65		0.18	0.60		0.18	0.60	
High risk (≤average)	38	1.68	2.45		0.21	0.58		0.05	0.23	
**Four Stage**	**46**	**1.37**	**2.27**	**0.37**	**0.22**	**0.59**	**0.99**	**0.09**	**0.35**	**0.99**
Low risk (Stages 3 or 4)	13	1.62	3.18		0.00	0.00		0.00	0.00	
High risk (Stages 1 or 2)	33	1.27	1.86		0.30	0.68		0.12	0.42	

## Discussion

Findings support the Community OEP is viable model when offered in a rural and underserved setting. The participants met the criteria for the OEP in that they demonstrated impairments in mobility and a history of falls. Though the attrition rate was quite high (75% for the 6-month intervention), those participants that completed the program demonstrated significant improvements in functional and self-report measures. This is one of the first translational studies to report outcomes from this innovative model in which the program is managed by an AAA, delivered by a COTA and a personal trainer, and overseen by a PT. The development of this model stemmed from the recognized need, and available resources, for an in-home evidence-based fall prevention program. These findings have vast implications for reach and dissemination of the OEP.

Baseline measures were collected for a total of 239 participants. On average, participants represented a group that was quite frail and at a high risk for experiencing a fall or fall-related injury. Participants were, on average, younger but frailer and reported more falls and fall-related injuries compared to other groups studied (12–16, 30, 31). The majority of participants (87%) reporting fear of falling, and 60% had experienced a fall in the previous 12 months, which is a higher rate of falls than typical for this age group (1). The average number of falls experienced by the group was 1.7 per year, and, of great concern, the group reported a high rate of emergency room and hospital visits due to a fall.

Participants clearly met the criteria for the OEP participation (age 80 and over, at risk for falling based on functional performance) (17). On average, their risk for falls based on functional assessments was elevated. For example, TUG test times of >12 s are indicative of a high risk for falls (32) and >20 s are indicative of decreased mobility (29). The average TUG test score for participants in this study was 26.2 s, with only 29 (13%) able to complete the TUG in <12 s.

Of the 239 participants, outcome measures at 6 months were collected on 62 participants. Examining participant characteristics differences between completers and non-completers offers limited insight about the high attrition rate. The groups at baseline showed no significant differences in any measures except for the Chair Rise and the amount of PT received prior to starting the program. A greater proportion of non-completers (*n* = 54) were able to achieve the gender and age-based normative value for the Chair Rise. This indicates these individuals had sufficient lower-body strength to help protect them against a fall. These individuals may have been too high functioning for the OEP, which specifically targets frail older adults. As a result, they may not have been challenged and felt they were not benefiting from the program, resulting in either stopping the program early or transitioning to a different program such as A Matter of Balance or Tai Chi for a greater challenge.

Non-completers received more PT visits for a longer duration prior to starting the OEP than the completers. This finding seems paradoxical; however, it may be that the perceived need for the OEP by those receiving PT was diminished because they were doing PT instead. It is quite common for patients of PT to discontinue their exercises once their therapy is complete, and this may have been the case for these particular participants (33). This finding warrants future investigation as does the high attrition rate.

Of the 62 completers, outcomes data were collected on 57. Self-reported measures of health status, satisfaction with physical activity levels, falls-related confidence, and functional ability improved significantly between baseline and 6 months. The largest changes were documented in confidence to prevent a fall with 42.9% of participants agreeing or strongly agreeing in this statement at baseline, and 80.4% at 6 months. Given the high number of falls among this population, this finding is quite compelling. Fear of falling can have a significant impact on quality of life and mobility status (21, 34, 35). Minimized fear of falling can result in increased activity engagement such as physical activity. Findings support the Community OEP may have reduced fear of falling based on participants increased confidence and their satisfaction with physical activity levels (increased from 17% at baseline to 54% at 6-month post-assessment). Significant improvements were reported for all mobility questions except for climbing stairs, which did increase from 9.1% at baseline to 20.0% at 6-month post-assessment. Even though climbing stairs is a prescribed activity in the OEP, it is not prescribed until the participant can safely perform the activity independently. In the current study, participants may have been too frail to climb stairs as part of the OEP and may not have been able to perceive this activity as a benefit after 6 months.

Improvements were recorded for all functional measures, with the greatest improvements in the ability to hold either Stage 3 (heel-toe position) or Stage 4 (standing on one leg position) of the Four Stage for at least 10 s. The inability to hold either of these positions for at least 10 s is linked to a higher risk of falling (27). The number of participants who achieved this position nearly doubled during the 6 months of the intervention. Improvements were reported for the TUG and Chair Rise; however, the majority of participants still scored in the “at-risk” category, and only 12 of the 57 completers were able to perform the TUG in less than 12 s.

The improvements in functional and self-perceived outcomes are similar to those reported in the literature. We have previously reported on an implementation study of the OEP by physical therapists in the US (36). Participants demonstrated an average improvement in TUG times of 2.8 s and an average increase of 1.75 on the Chair Rise after 8 weeks. Subjects in the current study demonstrated improvements of 4.36 s for the TUG and 1.88 for the Chair Rise after 6 months of participation. The current intervention is a longer intervention; however, the fact that the participants were more frail compared to the PT study supports the Community OEP can result in improved functional performance and decreased fall risk. In addition, the improvements in the Four Stage were similar to those reported by Campbell at 6 months in the original OEP study (12).

The population studied reported a high rate of falls and fall-related injuries. The current study was not powered to detect a change in falls or fall-related injuries. However, we were able to collect data on major events experienced over the course of the program to identify any key factors that may increase an individual’s risk of falling. Participants were categorized into high risk and low risk based on functional performance at baseline. Falls and fall-related visits to the ED and hospital admissions were recorded for the 6 months of the intervention. The only significant finding was that individuals who had greater lower extremity strength based on Chair Rise experienced significantly fewer falls than those with worse lower extremity strength. This finding was not replicated in number of ED visits and hospitalizations, but this is probably due to the low number of these events. This information supports the importance of lower extremity strength to protect against falls and fall-related injuries.

The length of an intervention is an important factor in the success of the program. This is especially true when programs like the OEP are implemented in “Fee-for-Service” settings like the US. The implementation of the OEP in the US has encountered many billing and reimbursement barriers (20). As a result, many PTs in the US limit the OEP to an 8-week intervention. However, for those older adults who are more impaired, an 8-week intervention may be too short to achieve optimal outcomes. The alternative delivery model posed by NWSDS was not funded through the Medicare Fee-For-Service model. Rather, it was funded by grants allowing for more flexibility in program delivery and a greater ability to replicate the frequency and duration of the program. The NWSDS model kept participants in the program longer and provided support similar to the original program without using a physical therapist. It may be that the critical component of the OEP is the long duration with the follow-up phone calls, and this question should be answered in future studies.

### Limitations

A limitation of this study was the lack of diversity among participants. As this was a translational study, the intervention was available to the population served by the AAA. The demographics for the study are consistent with a 2013 CMS report, which stated 95% of those receiving Medicare services were white. A second limitation is a lack of a comparison group. This was a translational study of a program as it was implemented by an AAA. Therefore, we were not able to randomize participants into intervention and control groups. The goal of this study was to determine the effectiveness of the program in a real-world setting. However, the OEP has been well studied in a variety of populations and a variety of settings (13, 15, 16, 37). Given this was a variation on an established model with evidence of effectiveness, there is ample opportunity to compare outcomes with published studies to determine the feasibility of this model for future studies. Another limitation of the study was the lack of ability to follow-up with those who did not continue for the duration of the intervention. The AAA was not resourced to follow those individuals who did not complete the program. They were only able to report on those individuals who were active participants in the OEP. Of the 259 who started the program, only 57 completed 6 months of the intervention. It is imperative to understand the key elements that motivated those 57 to continue for the duration and why the remaining 153 did not complete the program.

## Conclusion

Frail older adults are at a high risk for falling. This is a population that would most benefit from a tailored program delivered in the home with both face-to-face visits and telephone support. Using licensed professionals like physical therapists to deliver these types of programs can be resource intensive and potentially cost-prohibitive. Hybrid models that utilize the PT as an “as needed” consultant, licensed assistants, and fitness professionals may offer a viable, low-cost solution to deliver these types of programs to those who need it most. Results from this paper support that these types of models can result in improved outcomes for participants. More work needs to be done to investigate adherence and compliance to these types of programs and testing additional alternative delivery models.

## Author Contributions

TS was responsible for project design and dissemination, developing the database reviewing all data, writing up all aspects of the manuscript, and coordinating the work on the manuscript between the data collectors, the statisticians, and co authors. LG was responsible for organizing the program, working with the PTs to consult, working with the COTAs to implement, recruitment, and retention of participants. MS was responsible for data analysis and interpretation and assisted with paper review and content. LJ was responsible for all statistics. HR was responsible for delivering the intervention and all data collection. MO was responsible for project design and manuscript review.

## Disclaimer

The findings and conclusions in this article are those of the author(s) and do not necessarily represent the official position of the Centers for Disease Control and Prevention.

## Conflict of Interest Statement

The authors declare that the research was conducted in the absence of any commercial or financial relationships that could be construed as a potential conflict of interest.
